# Supplementary material for: Molecular characterization of Rft1, an ER membrane protein associated with congenital disorder of glycosylation RFT1-CDG
Source: J Biol Chem. 2024 Jul 16;300(8):107584. doi: 10.1016/j.jbc.2024.107584 (PMC11365447; doi:10.1016/j.jbc.2024.107584)
Supplement: Supporting Information [file mmc1.docx]

Supporting Information

Molecular characterization of Rft1, an ER membrane protein associated with congenital disorder of glycosylation RFT1-CDG

Eri Hirata^1‡^, Ken-taro Sakata^1‡^, Grace I. Dearden^1^, Faria Noor^1^, Indu Menon^1^, George N. Chiduza^2^, Anant K. Menon^1^*

^1^Department of Biochemistry, Weill Cornell Medical College, New York, NY 10065, USA

^2^Structure and Function of Biological Membranes - Chemistry Department, Université Libre de Bruxelles - Campus Plaine, 1050 Brussels, Belgium


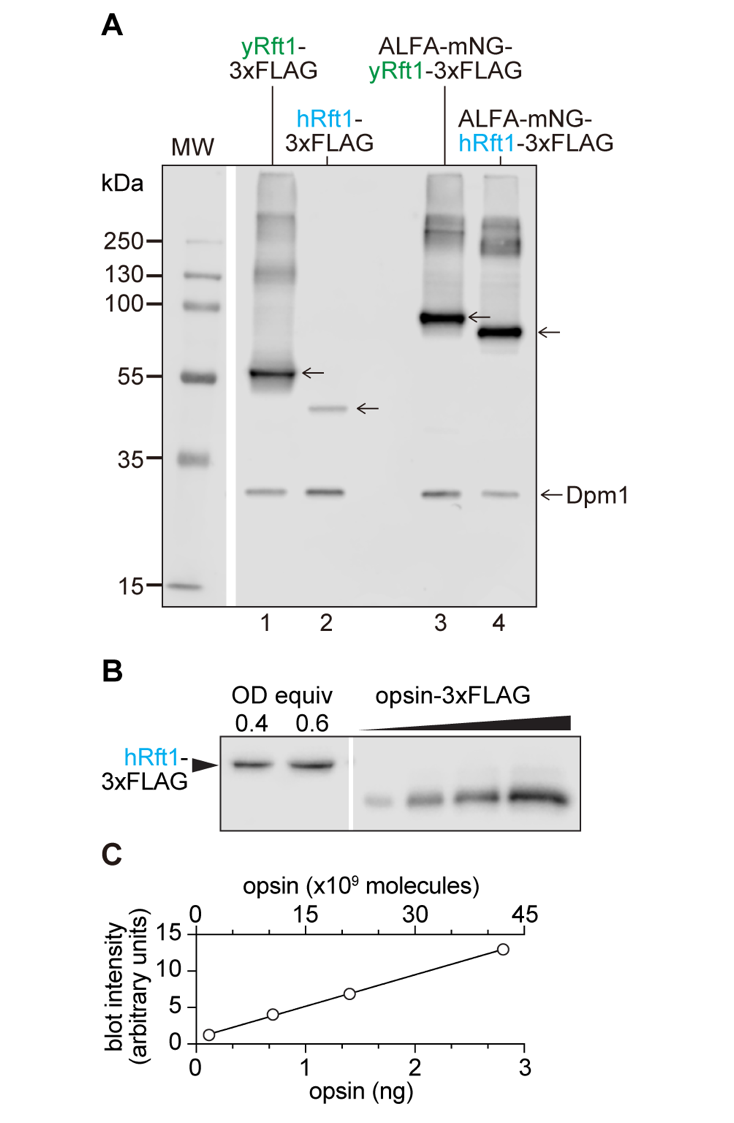


Supplementary Figure S1. Quantifying expression of hRft1 constructs.

**A.** Expression of yRft1 and hRft1 proteins. Plasmid shuffling was used to replace hRft1-3xFLAG (expressed from a *URA3* plasmid) in KSY512 cells with yRft1-3xFLAG, hRft1-3xFLAG, ALFA-mNG-yRft1-3xFLAG, and ALFA-mNG-hRft1-3xFLAG expressed from *HIS3* plasmids under control of the GPD promoter. Cells were cultured to log-phase, harvested, and analyzed by SDS-PAGE and immunoblotting with anti-FLAG and anti-Dpm1 (loading control) antibodies. Unmarked arrows indicate Rft1-3xFLAG and ALFA-mNG-Rft1-3xFLAG. All Rft1 constructs migrate faster than expected based on their predicted molecular weights; yRft1 is approximately 6 kDa larger than hRft1. The relative expression levels of yRft1 and hRft1 constructs is as follows (mean ± S.D., n=3 biological replicates): yRft1-3xFLAG/ hRft1-3xFLAG = 6.7 ± 2.4 and ALFA-mNG-yRft1-3xFLAG/ ALFA-mNG-hRft1-3xFLAG = 1.2 ± 0.5.

**B.** KSY512 cells expressing hRft1-3xFLAG were cultured to log-phase and proteins (corresponding to 0.4 and 0.6 OD equivalents of cells) were analyzed by SDS-PAGE alongside 3xFLAG-tagged bovine opsin standards (0.12, 0.70, 1.4, and 2.8 ng (obtained by diluting a 117 ng/µL stock solution)(Ploier et al. (2016) Nat Commun 7, 12832)). FLAG-tagged hRft1 and opsin were visualized by immunoblotting with anti-FLAG antibodies. The opsin band runs just above the 35 kDa molecular weight marker; hRft1 runs between the 55 and 35 kDa markers as seen in panel A, lane 2.

**C.** The immunoblot signal intensity for the opsin standards (obtained by densitometry using ImageJ) was graphed against the protein amount loaded onto the gel (bottom x-axis), and linear regression was used to obtain a calibration plot. The x-axis at the top of the plot was obtained by converting opsin mass to number of molecules using a molecular weight of 40,000 g/mole. This plot was used to convert the Rft1 signal intensity (3.8 and 5.1 units (obtained using ImageJ), corresponding to 0.4 and 0.6 OD equivalents, respectively) to number of molecules, yielding a final result of 840 ± 35 molecules/cell (n=2 technical replicates).
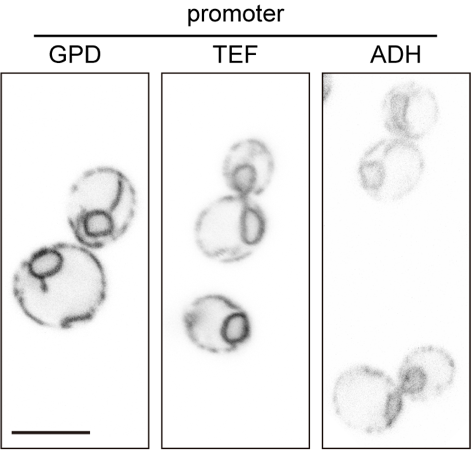


Supplementary Figure S2. Localization of mNG-hRft1 expressed under control of promoters of different strength.

The mNG-hRft1 construct was integrated into mCherry-HDEL-expressing wild-type cells, using three different promoters (GPD, TEF, ADH) to drive expression. The resulting cells (*P_GPD_*-mNG-hRft1 mCherry-HDEL, *P_TEF_*-mNG-hRft1 mCherry-HDEL, and *P_ADH_*-mNG-hRft1 mCherry-HDEL) were cultured in YPD medium to log-phase and imaged by confocal fluorescence microscopy to visualize mNG-hRft1. Images were taken under the same conditions, using the same microscope settings. Promoter strength GPD>TEF>ADH. Scale bar = 5 μm.


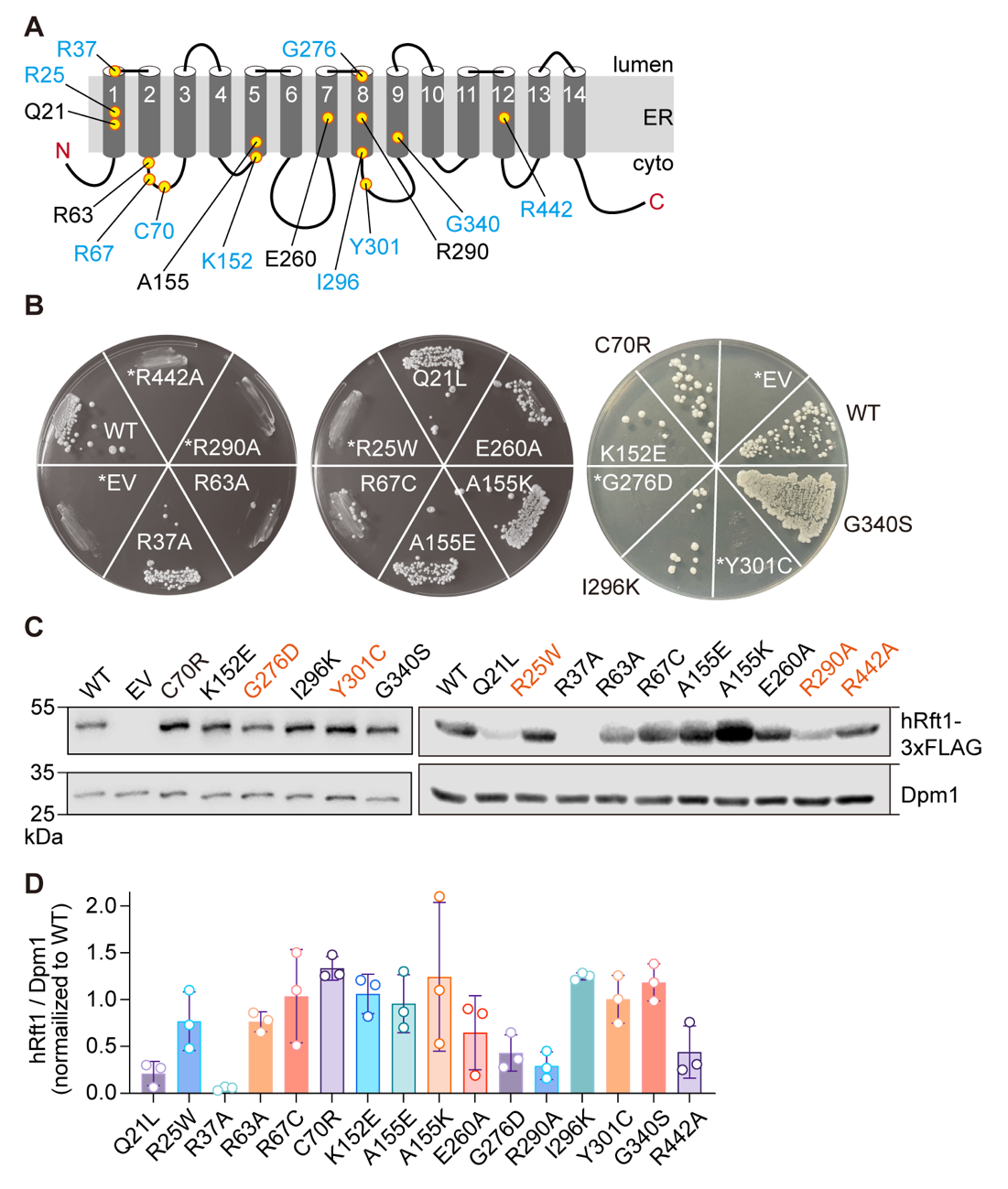


Supplementary Figure S3. Restriction of hRft1 to the nuclear ER does not affect function.

**A.** Topology model of hRft1 as shown in Figure 4A. Sites that were mutated in this study are indicated by yellow circles. Residues marked in blue correspond to Rft1-CDG mutations.

**B.** KSY512 cells (*rft1Δ*←hRft1(*URA3*)) were transformed with *HIS3* plasmids for expression of hRft1 point mutants with a C-terminal 3xFLAG tag. Colonies picked from SD(-Ura, -His) plates were streaked onto plates containing 5-FOA (1 mg/ml) to eject the *URA3* plasmid. Images were taken after 3 days of incubation at 30°C. The hRft1 mutants that did not grow on the 5-FOA plate are marked with an asterisk. EV, empty vector, WT, wild-type hRft1.

**C.** Wild type (BY4741) cells carrying *HIS3* plasmids for expression of hRft1 point mutants with a C-terminal 3xFLAG tag were cultured in SD(-His) medium and harvested. Immunoblots were done using anti-FLAG and anti-Dpm1 antibodies. Molecular weight markers are indicated on the left; tick marks on the right panels indicate the same markers.

**D.** hRft1-3xFLAG and Dpm1 (loading control) were quantified from panel C and two additional biological replicates. The ratio of the intensity of the hRft1 band to Dpm1 was calculated and normalized to that of the WT sample (mean ± S.D., n=3 biological replicates).


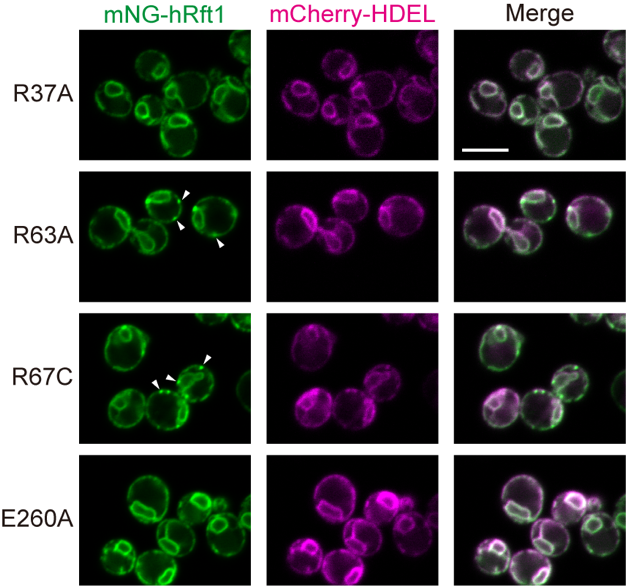


Supplementary Figure S4. Subcellular localization of hRft1 point mutants.

*P_GPD_*-mNG-hRft1^R37A/R63A/R67C/E260A^ mCherry-HDEL cells (YAKM248-251, Table 2) were cultured in YPD medium to log-phase and imaged by confocal fluorescence microscopy. The arrowheads indicate hRft1 varicosities seen in the cortical ER (2, 50, 54 and 25% of R37A, R63A, R67C and E260A-expressing cells, respectively (n>100 cells counted in each case) displayed these varicosities). Scale bar = 5 μm.
